# Comparing cross-sectional and longitudinal approaches to tuberculosis patient cost surveys using Nepalese data

**DOI:** 10.1093/heapol/czad037

**Published:** 2023-06-10

**Authors:** Daisy Bengey, Anchal Thapa, Kritika Dixit, Raghu Dhital, Bhola Rai, Puskar Paudel, Rajan Paudel, Govind Majhi, Tara Prasad Aryal, Manoj Kumar Sah, Ram Narayan Pandit, Gokul Mishra, Mukti Nath Khanal, Eliud Kibuchi, Maxine Caws, Noemia Teixeira de Siqueira-Filha

**Affiliations:** Department of Tropical Disease Biology, Liverpool School of Tropical Medicine, Pembroke Place, Liverpool L3 5QA, United Kingdom; Department of Clinical Sciences, Liverpool School of Tropical Medicine, Pembroke Place, Liverpool L3 5QA, United Kingdom; Birat Nepal Medical Trust, Lazimpat, Kathmandu, Ward No. 2, Nepal; Birat Nepal Medical Trust, Lazimpat, Kathmandu, Ward No. 2, Nepal; Department of Global Public Health, Karolinska Institutet, Stockholm 171 77, Sweden; Birat Nepal Medical Trust, Lazimpat, Kathmandu, Ward No. 2, Nepal; Birat Nepal Medical Trust, Lazimpat, Kathmandu, Ward No. 2, Nepal; Birat Nepal Medical Trust, Lazimpat, Kathmandu, Ward No. 2, Nepal; Birat Nepal Medical Trust, Lazimpat, Kathmandu, Ward No. 2, Nepal; Birat Nepal Medical Trust, Lazimpat, Kathmandu, Ward No. 2, Nepal; Birat Nepal Medical Trust, Lazimpat, Kathmandu, Ward No. 2, Nepal; Birat Nepal Medical Trust, Lazimpat, Kathmandu, Ward No. 2, Nepal; Birat Nepal Medical Trust, Lazimpat, Kathmandu, Ward No. 2, Nepal; Department of Clinical Sciences, Liverpool School of Tropical Medicine, Pembroke Place, Liverpool L3 5QA, United Kingdom; Birat Nepal Medical Trust, Lazimpat, Kathmandu, Ward No. 2, Nepal; Planning Monitoring Evaluation & Research Section, National Tuberculosis Control Center, Thimi, Bhaktapur, Nepal; MRC/CSO Social and Public Health Sciences Unit, School of Health and Wellbeing, University of Glasgow, 90 Byres Road, Glasgow G12 8TB, United Kingdom; Department of Clinical Sciences, Liverpool School of Tropical Medicine, Pembroke Place, Liverpool L3 5QA, United Kingdom; Birat Nepal Medical Trust, Lazimpat, Kathmandu, Ward No. 2, Nepal; Department of Health Sciences, University of York, Heslington, York YO10 5DD, United Kingdom

**Keywords:** Tuberculosis costing, longitudinal vs cross-sectional survey, tuberculosis and Nepal

## Abstract

The World Health Organization has supported the development of national tuberculosis (TB) patient cost surveys to quantify the socio-economic impact of TB in high-burden countries. However, methodological differences in the study design (e.g. cross-sectional vs longitudinal) can generate different estimates making the design and impact evaluation of socio-economic protection strategies difficult. The objective of the study was to compare the socio-economic impacts of TB estimated by applying cross-sectional or longitudinal data collections in Nepal. We analysed the data from a longitudinal costing survey (patients interviewed at three time points) conducted between April 2018 and October 2019. We calculated both mean and median costs from patients interviewed during the intensive (cross-sectional 1) and continuation (cross-sectional 2) phases of treatment. We then compared costs, the prevalence of catastrophic costs and the socio-economic impact of TB generated by each approach. There were significant differences in the costs and social impacts calculated by each approach. The median total cost (intensive plus continuation phases) was significantly higher for the longitudinal compared with cross-sectional 2 (US$119.42 vs 91.63, *P* < 0.001). The prevalence of food insecurity, social exclusion and patients feeling poorer or much poorer were all significantly higher by applying a longitudinal approach. In conclusion, the longitudinal design captured important aspects of costs and socio-economic impacts, which were missed by applying a cross-sectional approach. If a cross-sectional approach is applied due to resource constraints, our data suggest that the start of the continuation phase is the optimal timing for a single interview. Further research to optimize methodologies to report patient-incurred expenditure during TB diagnosis and treatment is needed.

Key messages

- Significantly different cost estimates and socio-economic impact of tuberculosis were found when comparing cross-sectional and longitudinal approaches. These differences can impact the implementation of public policies addressing the reduction of catastrophic costs and the provision of social protection.
- Despite being the best alternative, the implementation of a longitudinal cost survey can be burdensome, particularly for resource-constraint countries.
- The cross-sectional study design provides more accurate estimates when the interviews are performed during the initial stage of the continuation treatment phase.

## Introduction

Tuberculosis (TB) remains one of the top 10 causes of death worldwide (World Health Organization(WHO), 2021). Until Coronavirus disease, TB was the leading cause of death from a single infectious agent. Sadly, deaths from TB increased for the first time in a decade in 2021, with an estimated 1.5 million deaths in 2020 (World Health Organization, 2021). This burden is not uniform. Low- and middle-income countries (LMICs) accounted for 97% of all reported TB cases in 2019 (World Health Organization, 2019). Moreover, while TB can affect anyone, poorer individuals and those within resource-constrained settings are at a greater risk of developing the disease (Pedrazzoli *et al.*, 2019).

Despite the progress made by some countries towards the WHO End TB Strategy milestones, TB incidence has not fallen as rapidly as anticipated (Uplekar *et al.*, 2015; World Health Organization, 2021). This has been attributed in part to the economic barriers that limit patient access to health services (World Health Organization, 2015; Dixit *et al.*, 2019; Biermann *et al.*, 2021). Access to free TB diagnosis and treatment is now widely available in most LMICs. Despite this, TB patients still incur substantial costs and income loss while navigating the pathway of obtaining diagnosis and treatment (Pedrazzoli *et al.*, 2019; Marahatta *et al.*, 2020). Health-seeking behaviour of TB patients is also inhibited by economic barriers that contribute to delays in TB diagnosis and treatment (Tanimura *et al.*, 2014). The high out-of-pocket expenditure and indirect costs associated with TB treatment also further impoverish households, due to higher catastrophic costs, which can also increase food insecurity (Mauch *et al.*, 2011; Marahatta *et al.*, 2020; Dixit *et al.*, 2021).

To identify and quantify costs incurred by TB-affected families, national patient costing surveys have been carried out in several countries (Wingfield *et al.*, 2014; Pedrazzoli *et al.*, 2019). Costing surveys are an important tool to guide the creation of social protection policies to support TB patients and their families. Results from multiple national costing surveys have highlighted the urgent need to implement such strategies to mitigate the financial and social barriers of TB and facilitate access to TB diagnosis and treatment (Grede *et al.*, 2014; Nhung *et al.*, 2018).

Although TB patient costing surveys have provided valuable insights into patient-incurred costs, methodological differences in the study design (e.g. cross-sectional or longitudinal) have made comparison between countries and monitoring of progress difficult. In cross-sectional studies, data are collected on a single occasion during the patient treatment (intensive or continuation phase of treatment). Longitudinal studies collect data at several time points, usually three, throughout the treatment, particularly aiming to capture any differences between the prediagnostic phase, the intensive phase and the continuation phase of TB treatment. To address the problem of variations in methodology, a consortium of partners developed The Tool to Estimate Patients’ Costs to standardize data collection and analysis in 2008 (Rudgard *et al.*, 2017). The WHO Global TB programme later used the tool to develop a generic protocol and a costing questionnaire with further standardized and globally applicable methodology (KNCV Tuberculosis Foundation, World Health Organization and Japan Anti-Tuberculosis Association, 2008). This tool recommends a cross-sectional design with a single interview at any phase of treatment and extrapolation of the reported costs to estimate the total treatment costs, catastrophic costs and socio-economic impacts such as food insecurity and social exclusion (KNCV Tuberculosis Foundation, World Health Organization and Japan Anti-Tuberculosis Association, 2008).

Despite the methodological advantages of the cross-sectional-based WHO protocol, concerns have been raised about data accuracy and reliability because ‘crude extrapolation’ may not account for variations in the cost and socio-economic impact throughout the various phases of the disease treatment (World Health Organization, 2017). Therefore, several authors have suggested that a longitudinal approach would generate more accurate estimates (Evans *et al.*, 2021; Sweeney *et al.*, 2016; Nhung *et al.*, 2018; Gurung *et al.*, 2019; 2021). Additionally, this approach would potentially identify the time points when patients are most likely to incur high costs, guiding the optimal design of social protection policies to mitigate the economic impact of TB (Gurung *et al.*, 2019). However, longitudinal studies are also considerably more expensive, labour intensive and burdensome to the TB-affected households, and therefore, evidence is required to justify their application (KNCV Tuberculosis Foundation, World Health Organization and Japan Anti-Tuberculosis Association, 2008).

We analysed a longitudinal costing study conducted in Nepal to compare the cost estimates calculated through the cross-sectional and longitudinal designs. We aimed to determine if the additional costs and complexity of the longitudinal approach can be justified. We also compared the estimates generated if the cross-sectional survey was conducted in the intensive or continuation phase to determine the impact of the timing of the cross-sectional interview on cost estimates.

## Methods

### Study setting

Nepal is an LMIC in South Asia, bordered by India and China. With a population of 30 million people, the country has a gross domestic product per capita of US$1208 (World Bank, 2023). The country has 15% of the population living in extreme poverty (less than US$1.9 a day) (World Bank, 2010). Nepal was included in the WHO list of 30 countries with high multidrug-resistant/rifampicin-resistant-TB burden in 2021 after a national survey found a TB prevalence of 416/100 000 population [confidence interval (CI): 314–518], ∼1.6 times higher than previously estimated (Government of Nepal, 2020). The cost survey was implemented in four districts of Nepal: Dhanusha and Mahottari in Madhesh Province and Makwanpur and Chitwan in Bagmati Province. These provinces notified 17 813 TB cases in 2021/2022 (Government of Nepal *et al.*, 2023).

Since 1995, the Government of Nepal provides TB treatment free of charge in all health facilities up to the peripheral level (health posts) (Government of Nepal, 2019; Marahatta *et al.*, 2020). The country follows the TB management guidelines, which recommends directly observed treatment short-course (DOTS) for drug-sensitive TB as follows: 2 months of intensive phase followed by 4 months of continuation phase. Hospitalization is recommended only in case of severe adverse events or other complications during the treatment (Government of Nepal, 2019).

### Study population

New and relapsed drug-sensitive TB patients (*n* = 221) aged ≥18 years were recruited between April 2018 and October 2019.

### Study design

**Figure 1.**
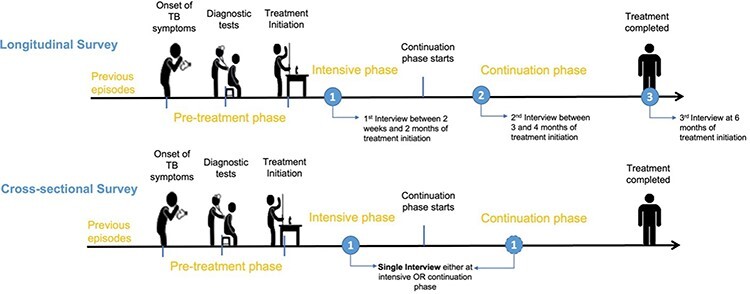
The timing of interviews by the methodological approach

We analysed the data from a longitudinal patient costing survey, which was a component of the European Horizon–funded IMPACT TB project (www.impacttbproject.org). The WHO TB patient costing tool was adapted to the local context, with appropriate terminology for the local health system structure and household assets. The adapted survey was then translated and validated with 10 sample interviews. Trained community health workers conducted the survey to collect socio-economic impact (e.g. food insecurity and social exclusion) and direct medical (e.g. drugs, tests and medical fees), non-medical (e.g. transportation and food) and indirect (e.g. time and income loss) costs. The longitudinal approach included all the costs incurred by the patient throughout the pretreatment and treatment periods as indicated later and in Figure 1:

- The first interview was conducted between 2 weeks and 2 months of the intensive phase and collected data on patient costs incurred since the onset of TB symptoms until treatment initiation (pretreatment costs) in addition to costs incurred from treatment initiation until the date of the first interview (treatment costs).
- The second interview was conducted during the continuation phase between third and fourth months of treatment and collected costs incurred since the first interview until the date of the second interview. This interview included costs incurred in the intensive and continuation phases.
- The third interview was administered at the end of the sixth-month treatment and collected costs incurred in the continuation phase since the date of the second interview.

#### Patient costs

Direct medical and non-medical costs were self-reported by the participants. Indirect costs were calculated according to the human capital approach (Pearce, 2016) using the self-reported time spent absent from work, the 2018 Nepali monthly minimum wage (US$12 105) (Government of Nepal and Ministry of Labor, 2018), the labour force participation rate (49%) (World Bank, 2018) and the unemployment rate (1.2%) (World Bank, 2018). The hourly (US$0.62) and daily (US$4.67) minimum wages were used to convert the time lost seeking diagnosis and care into monetary value. Costs were collected between April 2018 and October 2019 in local currency, Nepalese Rupees (NPR), and converted into US$, applying the average exchange rate from OANDA during the data collection period (NPR 1 = US$0.009) (https://www.oanda.com).

#### Adaptation of the longitudinal approach

**Figure 2.**
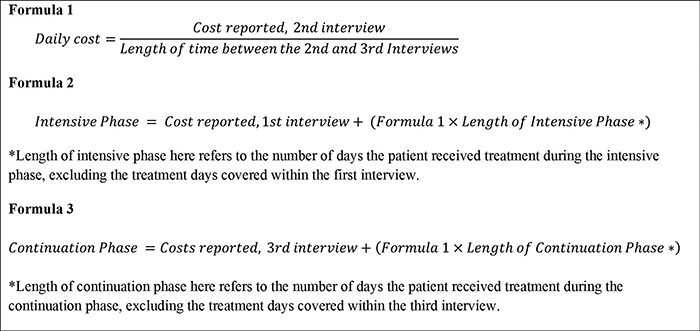
The formula of the longitudinal approach

The longitudinal approach estimates the total treatment costs rather than the cost per intensive or continuation phase of treatment. Therefore, to obtain the costs of each treatment phase, we separated the costs reported during the second interview into intensive and continuation phases by calculating the daily costs (Formula 1, Figure 2). We then determined the cost of the intensive and continuation phases incurred between the first and second interviews by multiplying the daily costs by the number of days referring to the intensive and continuation phases. The total cost of the intensive phase was determined by summing the costs reported in the first interview and the intensive phase cost estimates calculated from the second interview (Formula 2, Figure 2). The total continuation phase costs were calculated by summing the costs reported in the third interview and the continuation phase cost estimates calculated from the second interview (Formula 3, Figure 2).

#### Cross-sectional approach

**Figure 3.**
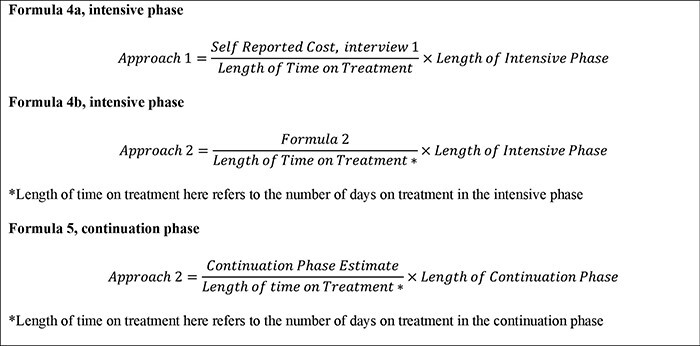
The formula of the cross-sectional approach

As shown in Figure 1, in the cross-sectional survey design, a single interview is conducted during the treatment phase. For the purpose of the study, to determine the appropriate timing of a single interview, we evaluated two different time points for the cross-sectional interview. In the first approach, the data from the first interview were used to extrapolate the intensive phase costs (cross-sectional 1). We referred to the WHO TB Patient Cost Handbook to extrapolate the costs, i.e. if one-third of the treatment phase is complete, costs can be extrapolated by multiplying costs to date by 3 (World Health Organization, 2017). Cross-sectional 1 did not estimate costs incurred during the continuation phase of treatment as we cannot extrapolate these costs to the continuation phase of treatment. In the second approach, we used the data from the second interview to extrapolate the intensive and continuation phase costs (cross-sectional 2) (Formulas 4a, 4b and 5, Figure 3). We then compared the cost estimates obtained from applying each approach to understand the impact of the timing of the cross-sectional interview on the cost estimates obtained.

### Catastrophic costs

We applied the WHO’s definition of TB-related catastrophic costs defined by the total cost incurred by patients exceeding the 20% threshold of the household self-reported annual pre-TB income (World Health Organization, 2014). We did not include pretreatment cost, as recommended by the WHO, in the calculation of catastrophic costs, as longitudinal and cross-sectional 1 produce the same estimates and cross-sectional 2 does not estimate pretreatment costs.

### Socio-economic impact

We compared the proportion of self-reported socio-economic impact in terms of food insecurity, social exclusion and sense of relative economic status (e.g. feeling poorer or much poorer) generated by each methodological approach.

### Statistical analysis

The datasets were analysed using the International Business Machines Statistical Package for the Social Sciences Statistics Data Editor Version 24. We calculated mean costs with 95% CI and median costs with the interquartile range (IQR) for each cost category, TB treatment phase and methodological approach. The difference in cost estimates calculated through the cross-sectional and longitudinal approaches was compared using the Wilcoxon signed-rank test. The prevalence of catastrophic costs and socio-economic impact was compared using the *t*-test. *P*-values ≤0.05 were considered statistically significant.

## Results

### Patient characteristics and TB treatment characteristics

More than half of the participants were male (67%), and the mean age was 48 years. Most patients reported low education levels, with 85% reporting no education or completion of primary/lower secondary level (from 1 to 8 years of education). The asset ownership more frequently reported was mobile (92%), livestock (71%), bicycle (66%) and television (56%) (Table 1). Table 1 also summarizes the treatment characteristics of the patients included in the study. The median number of weeks between the onset of TB symptoms and the treatment initiation was 6 weeks for the patients, and the average number of visits to the health provider during the pretreatment period was 3.7 and the treatment period was 2.2.

**Table 1.** Baseline socio-economic and treatment characteristics of TB patients, Nepal 2019

Socio-economic characteristics	*n* (%)
Sex, *n* (%)	
Male	147 (67)
Age, mean (SD)	48 (16)
Completed education, *n* (%)^a^	
No education	146 (66)
Basic school	42 (19)
Secondary school	33 (15)
Occupation, *n* (%)	
Farmer	39 (18)
Manual labour	47 (21)
Unemployed	60 (27)
Others	75 (34)
Patient income quartile, *n* (%)	
Poorest	94 (43)
Moderately poor	19 (9)
Average	54 (24)
Wealthiest	54 (24)
Household income quartile, *n* (%)	
Poorest	69 (31)
Moderately poor	44 (20)
Average	58 (26)
Wealthiest	50 (23)
Source of drinking water, *n* (%)	
Piped	74 (33)
Others	147 (67)
Toilet facilities, *n* (%)^b^	
No toilets	41 (19)
Public sewerage	6 (3)
Others	173 (79)
Access to electricity, *n* (%)	202 (93)
Assets, *n* (%)	
Mobile/phone	200 (92)
Refrigerator	31 (14)
Television	122 (56)
Radio	76 (35)
Bicycle	144 (66)
Motorbike	44 (20)
Livestock	156 (71)
**Treatment characteristics**	***n* (%), mean (SD)**
Treatment status, *n* (%)	
New	214 (97)
Retreatment and relapse	7 (3)
HIV status, *n* (%)	
Positive	2 (1)
Negative	153 (69)
Unknown	66 (30)
Hospitalization during pretreatment^c^, *n* (%)	28 (13)
Hospitalization during treatment, *n* (%)	3 (1)
Number of visits to health providers, pretreatment^c^, mean (SD)	3.7 (2.2)
Type of service visited^d^, *n* (%)	
Public health centres/hospitals	413 (52)
Private clinics/hospitals	213 (27)
Others^e^	172 (21)
Number of visits to health providers, treatment, mean (SD)	2.2 (1.3)
**Treatment characteristics**	*n* (%)
Type of services visited^f^, *n* (%)	
Public health centres/hospitals	411 (87)
Private clinics/hospitals	26 (5)
Others^d^	36 (8)

Total sample = 221.

a Basic schools = primary level/lower secondary level (1–8 years of education).

b One missing data.

c Seven patients were excluded from the analysis.

d One visit was missed.

e NGOs and informal providers such as pharmacists and traditional healers.

f Thirteen missing data.

HIV, human immunodeficiency virus; SD, standard deviation.

### Patient costs

Table 2 presents cost estimates according to the methodological approach by phase of treatment and cost category. For the intensive phase of TB treatment, cross-sectional 1 had the highest estimates (median US$56.35; IQR 21.72–108.34), followed by longitudinal (median US$50.86; IQR 19.98–92.65) and cross-sectional 2 (median US$31.28; IQR 12.51–98.87). The differences in estimates were statistically significant when comparing cross-sectional 1 vs cross-sectional 2 (*P* < 0.001), cross-sectional 1 vs longitudinal (*P* < 0.001) and cross-sectional 2 vs longitudinal (*P* < 0.001).

**Table 2.** Costs (US$) incurred by TB patients throughout treatment by methodological approach

	Cross-sectional approach 1^a^		Cross-sectional approach 2^b^		Longitudinal approach	
Treatment phase	Mean (95% CI)	Median (IQR)	Mean (95% CI)	Median (IQR)	Mean (95% CI)	Median (IQR)
Intensive phase						
Direct medical	24.39 (13.48–35.31)	7.54 (3.23–18.76)	4.04 (3.46–4.62)	2.66 (1.46–4.56)	12.40 (8.42–16.37)	5.28 (2.74–10.97)
Direct non-medical	6.46 (4.80–8.12)	2.23 (0.00–6.15)	4.41 (3.16–5.65)	0.00 (0.00–4.50)	5.20 (4.04–6.37)	1.89 (0.25–5.40)
Indirect	54.32 (45.94–62.71)	32.74 (11.21–81.17)	45.22 (39.03–51.40)	18.05 (6.60–87.86)	47.03 (40.96–53.10)	30.62 (11.05–74.51)
Total	85.18 (69.41–100.95)	56.35 (21.72–108.34)	53.67 (47.19–60.15)	31.28 (12.51–98.87)	64.63 (56.69–72.57)	50.86 (19.98–92.65)
Continuation^c^ phase						
Direct medical	Not estimated	Not estimated	8.06 (6.90–9.22)	5.33 (2.92–9.12)	7.92 (6.60–9.24)	5.35 (3.18–9.23)
Direct non-medical	Not estimated	Not estimated	8.68 (6.29–11.07)	0.00 (0.00–9.00)	3.36 (2.46–4.25)	0.00 (0.00–3.43)
Indirect	Not estimated	Not estimated	90.08 (77.79–102.37)	36.11 (13.07–175.72)	91.64 (79.65–103.63)	36.87 (16.74–179.22)
Total	Not estimated	Not estimated	106.83 (93.97–119.68)	61.08 (25.02–197.73)	102.92 (90.80–115.04)	59.51 (26.57–184.53)
Intensive and continuation phase	Not estimated	Not estimated	160.49 (141.16–179.82)	91.63 (37.53–296.60)	167.55 (149.65–185.45)	119.42 (51.98–275.99)

a Patient costs extrapolated from the data provided in the first interview.

b Patient costs are extrapolated from the data provided in the second interview.

c Continuation phase estimates for the cross-sectional approach 1 are not displayed as the first interview collected intensive phase costs only.

Cost estimates for the continuation phase of TB treatment showed that cross-sectional approach 2 had a higher estimate (median US$61.08; IQR 25.02–197.73) compared with the longitudinal approach (median US$59.51; IQR 26.57–184.53). However, the difference was not statistically significant.

For the total median cost of treatment, cross-sectional approach 2 reported a lower estimate than the longitudinal approach (median US$91.63; IQR 37.53–296.60 vs median US$119.42; IQR 51.98–275.99, *P* < 0.001).

Analysing the data by cost category (direct medical, non-medical and indirect costs) during the intensive phase of treatment, cross-sectional 1 consistently produced higher median estimates compared with cross-sectional 2 and the longitudinal for direct medical costs (median US$7.54 vs median US$2.66 vs median US$5.28, all *P* < 0.001), direct non-medical costs (median US$2.23 vs median US$0.00 vs median US$1.89, all *P* < 0.001) and indirect costs (median US$32.74 vs median US$18.05 vs median US$30.62, all *P* < 0.001).

**Figure 4.**
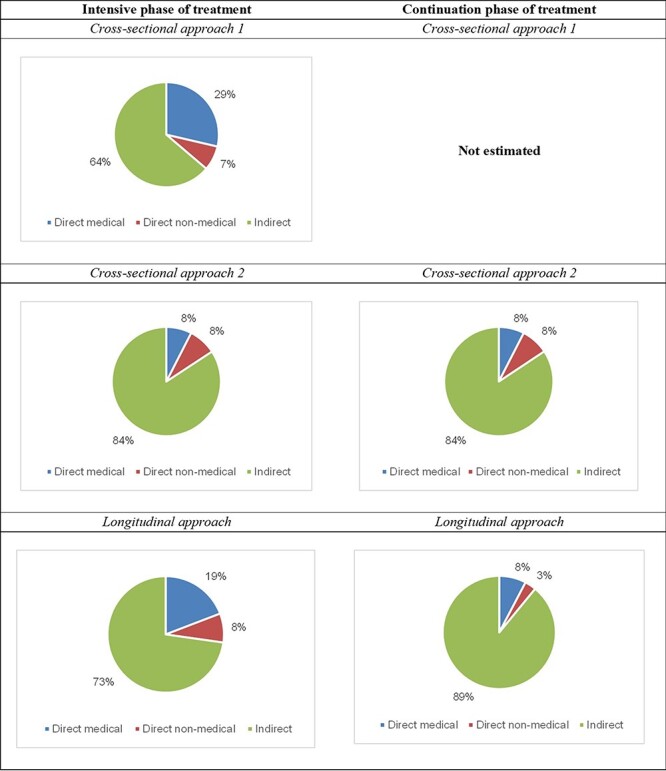
The proportion of each cost category by treatment phase and methodological approach

Despite the differences in cost estimates, all approaches reported that the indirect costs contributed on average two-thirds to the total costs of the intensive and continuation phases. Furthermore, all approaches showed that direct non-medical costs were the lowest contributor at <10%. Figure 4 shows the proportion of each cost by treatment phase and methodological approach.

### Catastrophic costs and socio-economic impact

**Figure 5.**
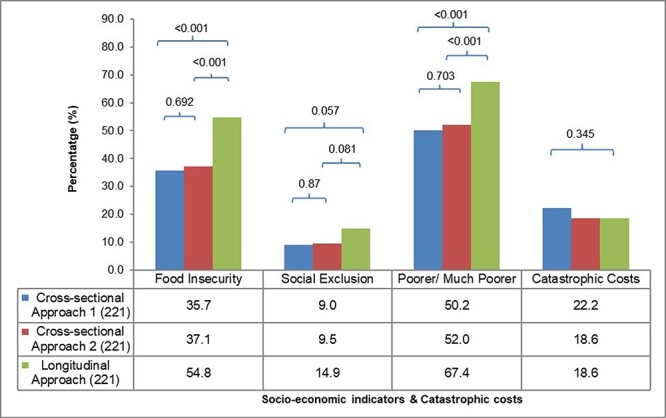
The socio-economic impact

Regardless of the approach, the percentage of TB patients facing catastrophic costs were consistently high. As shown in Table 3 and Figure 5, the prevalence of catastrophic costs was similar among the methodological approaches, varying from 18.6% for the longitudinal and cross-sectional 2 to 22.2% for cross-sectional 1.

**Table 3.** Prevalence of catastrophic costs by methodological approach

Variables	Cross-sectional approach 1	Cross-sectional approach 2	Longitudinal approach
District (*n*, %)			
Dhanusha	14 (26.4)	13 (24.5)	11 (20.8)
Mahottari	10 (16.7)	9 (15.0)	10 (16.7)
Makwanpur	16 (29.1)	13 (23.6)	14 (25.5)
Chitwan	9 (17.0)	6 (11.3)	6 (11.3)
Household income quartile^a^ (*n*, %)			
Poorest	38 (54.3)	33 (47.1)	36 (51.4)
Moderately poor	5 (11.4)	6 (13.6)	3 (6.8)
Average	5 (8.8)	2 (3.5)	2 (3.5)
Wealthiest	1 (2)	0 (0)	0 (0)
Total	49 (22.2)	41 (18.6)	41 (18.6)

a Based on self-reported household income before the TB episode.

The longitudinal approach registered a higher proportion of patients reporting socio-economic impacts compared with both cross-sectional approaches. However, the proportion of patients reporting socio-economic impact was similar between cross-sectional 1 and 2. The proportion of patients reporting food insecurity varied from 35.7% for cross-sectional 1 to 54.8% for the longitudinal. The proportion of social exclusion registered by the longitudinal approach was 1.5 times higher than cross-sectional 1 and 2. The proportion of patients with a self-reported sense of economic vulnerability was also higher in the longitudinal approach compared with cross-sectional 1 and 2.

## Discussion

This is the first direct comparison of cost estimates and socio-economic impact obtained by using either the longitudinal or cross-sectional approaches from a TB patient costing survey in Nepal. We found significant differences among the approaches for the intensive phase of treatment and total costs and proportion of self-reported socio-economic impacts. The prevalence of catastrophic costs was high regardless of the approach used, ranging from 19% to 22%. This reinforces an urgent need for effective financial protection schemes for TB patients to prevent them from being entrapped in the vicious cycle of poverty.

The estimates for the overall costs of treatment, cost of each treatment phase and type of cost (e.g. direct medical, direct non-medical and indirect) varied substantially depending upon the study design, which highlights the importance of further methodological development for patient costing surveys. Such surveys will always be subject to substantial recall bias, particularly in low-income countries where the informal economy is dominant. However, understanding the methodological limitations of different approaches and standardizing the methodology between settings can improve the accuracy of imperfect methods.

Cross-sectional approach 1, using the data from the interview conducted during the first 2 weeks of treatment, consistently produced the highest cost estimates, which were significantly greater for direct medical, direct non-medical and indirect. The mean total intensive phase cost estimates produced by this approach were ∼1.6 times higher than the estimate generated by the longitudinal approach and 1.8 times higher than cross-sectional approach 2, which used the interview conducted between 2 weeks and 2 months of treatment initiation. This suggests that single interviews conducted during the intensive phase of treatment may overestimate total costs, but this needs to be validated by further evaluations.

In our study, cost estimates for the continuation phase using a cross-sectional as compared with the longitudinal approach were not significantly different. This suggests less variation in costs during this phase. The cross-sectional approach is, therefore, more likely to provide reliable cost estimates when interviews are conducted between the third and fourth months of TB treatment. Hence, the extrapolation of costs may generate accurate cost estimates for this phase.

Both cross-sectional and longitudinal approaches produced higher costs in the continuation phase compared with the intensive phase. This contradicts the findings by Foster *et al.* (2015) who concluded from their prospective cohort study that costs were highest during the intensive phase and Tanimura *et al.* (2014) who concluded that prediagnosis costs and those incurred during the first 2 months of treatment dominate the treatment costs from their systematic review. However, our findings are consistent with a cross-sectional survey conducted in Tajikistan (Ayé *et al.*, 2010). The authors of this study suggest that the higher costs in the continuation phase are likely due to the accumulation of costs over a longer period as the continuation phase lasts for 6 months. However, the same study found that the monthly costs were significantly lower in the continuation phase when compared with the intensive phase (Ayé *et al.*, 2010). The cost of different treatment phases might vary by country with some settings presenting higher costs during the intensive phase and others in the continuation phase. Factors such as the accessibility of health services, source of income, broader social protection schemes and integration of TB services into primary care can all influence the patient pathway and resulting costs. There is also often a large difference between access to health services in rural and urban areas within countries, which can further influence the cost variability. Therefore, it is important to understand the local context before designing locally effective socio-economic support for TB-affected families.

When examining the cost categories, it is evident that the indirect costs dominate both treatment phases, ranging from 64% to 89% depending upon the approach and treatment phase. This is consistent with reports from other cross-sectional studies of different countries (Ananthakrishnan *et al.*, 2012; Mauch *et al.*, 2011; Nhung *et al.*, 2018). Tanimura *et al.* (2014) reported in a systematic review that the indirect costs ranged from 16% to 94% (unweighted average of 60%). Another systematic review of costing studies conducted in India reported that indirect costs contributed to 85% of the postdiagnosis cost. The review included 10 studies. However, the authors did not report the methodological approach adopted by the included studies. The review found that 12% of TB patients under DOTS treatment in Jalandhar, India, lost >60 days of work. The authors recommend the provision of food subsidies and social security schemes for those patients unable to continue with their jobs (Chandra *et al.*, 2020).

Our study found that all three approaches can generate similar estimates of catastrophic costs. Catastrophic cost is an indicator that reflects the economic hardships faced by people due to their illnesses. It shows that TB can trigger the medical poverty trap with severe long-term consequences for families, such as withdrawing children from education, loss of housing or losing essential income-generating assets. Catastrophic cost prevalence is one of the major indicators for the WHO End TB strategy, and it is important to obtain accurate measurements. Sweeney *et al*., (2020) suggested an alternative approach to estimating the national prevalence of CHE and uncertainties around those estimates through a deterministic cohort model. However, there remains a need for standardized methods to collect income data and report cost estimates to parameterize such models (Sweeney *et al.*, 2020).

Our study shows that several important socio-economic impacts of TB are underestimated by a cross-sectional approach. Importantly, the proportion of households reporting food insecurity, social exclusion and feeling poorer or much poorer were consistently higher in the longitudinal approach compared with either cross-sectional approach. The socio-economic impact can intensify throughout the lengthy treatment for TB, and this is only captured by a longitudinal study design. This demonstrates that the longitudinal costing surveys can reflect more accurately how the socio-economic status of a household has been affected and that important variations in these indicators can be lost when the cross-sectional approach is applied (Gurung *et al.*, 2021). Income changes are another important indicator of socio-economic impact, which cannot be captured by a cross-sectional survey. Different patterns have been reported in the literature, with some countries reporting a complete recovery of income lost by the end of treatment (Vo *et al.*, 2021) and others reporting a significant reduction in income during the intensive phase and no recovery by the end of the treatment (Gurung *et al.*, 2021). Clearly understanding the aspects and intensity of socio-economic impact is essential for planning social protection policies and determining the duration of such protective mechanisms in relation to TB treatment.

### Limitations

These specific findings can only be applied to the context of Nepal and cannot be generalized to other contexts. However, our analysis clearly shows a need for methodological studies to understand the optimal design and limitations of TB patient costing survey design. The validity of the self-reported cost data is subject to recall bias and the differences between real and perceived socio-economic impact. However, receipts and documentation for out-of-pocket expenditures are not common in rural Nepal, and income is often generated through the informal economy; therefore, there is no way to validate such reported expenditures. This study also underestimates the prevalence of catastrophic costs among Nepali people affected by TB because we did not include prediagnostic costs, which are known to be an important contributor to the total patient-incurred costs due to TB illness.

Socio-economic recovery is not instantaneous. Expenses occurring after the treatment completion but because of TB, such as funeral costs for patients who do not recover, are not reported. To inform the design of effective social and financial protection schemes, these costs also need to be accounted for.

## Conclusions

The longitudinal approach appears to capture the cost variations more accurately along the course of TB treatment than a cross-sectional approach. The longitudinal design can provide valuable insights to design effective social and financial protection schemes to support TB-affected households. However, in resource-constrained settings, conducting national longitudinal costing surveys may not be feasible because they are resource intensive to implement at a national level. In such cases, cross-sectional studies can be conducted with consideration given to the timing of the interview. A single interview conducted at the beginning of the continuation phase appears to be the best cross-sectional approach to estimate the total treatment costs associated with TB. The use of correction factors may be an alternative approach to improve the accuracy of estimates, but such approaches need careful validation. Finally, the longitudinal approach is necessary to understand the full scope and intensity of socio-economic impacts faced by TB-affected families. Important progress has been made in documenting the financial impact of TB on vulnerable households in the last decade. To maintain momentum and ensure the implementation of effective mitigation policies, it is important to further refine the methodology. Progress towards the End TB strategy goal of ‘zero TB-affected households suffering catastrophic costs by 2020’ has been dismal; to ensure we reach this goal by 2035, we must improve the monitoring and intensify political commitment.

## Data Availability

The data underlying this article cannot be shared publicly due to data protection law. The data will be shared on reasonable request to the corresponding author.

## Abbreviations

CHW = community health worker

CI = confidence interval

DOTS = directly observed treatment short-course

IBM SPSS = International Business Machines Statistical Package for Social Sciences

IQR = interquartile range

LMIC = low- and middle-income countries

MDR/RR = multidrug resistant/rifampicin resistant

NPR = Nepalese Rupees

TB = tuberculosis

US = United States

WHO = World Health Organization

## Reflexivity statement

This manuscript is a result of a collaborative research effort between the first author (D.B.) and the corresponding author (N.T.d.S.-F.) and the organization called Birat Nepal Medical Trust in Nepal. Data collection was mainly done by the authors in Nepal, and the analysis was conducted jointly by other authors. The first author is a female of British nationality, but most of the rest of the authors belong to the LMIC setting. The corresponding author is of Latin American origin and is currently based in the UK. All the authors who have the majority contribution to the paper are females (D.B., A.T., K.D., N.T.d.S.-F. and M.C.).
